# Biochemical characterization of a functional recombinant aryl-alcohol dehydrogenase from *Taiwanofungus camphorata*

**DOI:** 10.1186/1999-3110-55-14

**Published:** 2014-02-02

**Authors:** Chuian-Fu Ken, Che-Chi Chang, Lisa Wen, Jenq-Kuen Huang, Chi-Tsai Lin

**Affiliations:** 1 Institute of Bioscience and Biotechnology and Center of Excellence for the Oceans, National Taiwan Ocean University, 2 Pei-Ning Road, Keelung, 202 Taiwan; 2 Institute of Biotechnology, National Changhua University of Education, Changhua, Taiwan; 3 Department of Chemistry, Western Illinois University, 1 University Cir, Macomb, IL 61455 USA

**Keywords:** *Taiwanofungus camphorata*, Aryl-alcohol dehydrogenase, Three-dimension structural model (3-D structural model), Veratraldehyde, 3, 4-Dimethoxybenzyl alcohol, 2, 4-Dimethoxybenzyl alcohol

## Abstract

**Background:**

Aryl-alcohol dehydrogenases (AADs) have been known to involve in the metabolism of aromatic compounds.

**Results:**

One TcAAD cDNA (GenBank HQ453361) encoding a putative aryl-alcohol dehydrogenase (AAD) was cloned from *Taiwanofungus camphorata*. The deduced amino acid sequence is conserved among the reported AADs. A 3-D structural model of the TcAAD has been created based on the known structure of voltage-dependent potassium channels subunit beta-2 (PDB code: 3EAU). To characterize the TcAAD, the coding region was subcloned into an expression vector and transformed into *Saccharomyces cerevisiae*. The recombinant His6-tagged TcAAD was overexpressed and purified by Ni affinity chromatography. The purified enzyme showed a band of approximately 39 kDa on a 12% SDS-PAGE. The molecular mass determined by MALDI-TOF is 40.58 kDa which suggests that the purified enzyme is a monomeric enzyme. Using veratraldehyde as a substrate, the *K*_M_, V_max_ of TcADD was determined at pH 6.0. Using benzyl alcohol derivatives as substrates, the oxidizing power of TcADD via NAD^+^ at pH 9.6 was studied.

**Conclusions:**

The coding sequence of the TcAAD cDNA was introduced into an *S. cerevisiae* expression system and the active enzyme purified and characterized. Understanding the properties of this TcAAD will be beneficial for its potential in xenobiotic detoxification or production of natural flavors.

**Electronic supplementary material:**

The online version of this article (doi:10.1186/1999-3110-55-14) contains supplementary material, which is available to authorized users.

## Background

*Taiwanofungus camphorata* (*T. camphorata,* formerly named *Antrodia camphorata*) is a high valued mushroom found only in the forests of Taiwan. It has been used for centuries as health food and drug intoxication, among others (Ao et al., 2009). *T. camphorata* has been shown to exhibit anti-inflammatory properties (Hsieh et al., 2010). The compounds identified from the fruiting bodies or mycelia of *T. camphorata* in a submerged culture are benzenoids, diterpenes, maleic and succinic acid derivatives, polysaccharides, steroids, and triterpenoids (Ao et al., 2009). A lot of studies were aimed to find the exact bioactive compounds of the mushroom (Ao et al., 2009). In order to search for physiologically active components including antioxidant enzymes, we have established an expressed sequence tag (EST) from the fruiting bodies of *T. camphorata.* Based on the established EST*,* several antioxidant enzymes including a superoxide dismutase (Liau et al., 2007), a 1-Cys peroxiredoxin (Wen et al. 2007), a catalase (Ken et al., 2008), a glutathione formaldehyde dehydrogenase (Huang et al., 2009), a dithiol glutaredoxin (Ken et al., 2009), a 2-Cys peroxiredoxin isozyme (Liau et al., 2010) and a monothiol glutaredoxin (Ken et al., 2011) have been cloned and characterized. This encourages us further to search for active components from the established EST of *T. camphorata* for potential health food applications.

Aryl-alcohol dehydrogenases (AADs) have been known to involve in the metabolism of aromatic compounds (Muheim et al., 1991). Fungal AADs are also believed to be involved in the biosynthesis of veratryl alcohol as a secondary metabolite by white-rot fungi (Jensen et al., 1994; Yang et al., 2012). The glycosylation reaction is one of the major fungal reactions for xenobiotic detoxification (Jensen et al., 1994) and the formation of hydroxymethyl group is essential for glycosylation reactions. AAD may play an important role in maintaining a hydroxymethyl group under oxidative circumstances for effective xylosylation reaction (Ichinose et al., 1999; Ichinose et al., 2002) on the microbial production of natural flavors and fragrances such as the rose-like flavor compound of 2-phenylethanol (Yang et al., 2012).

Here, we report the cloning, expression and purification of a functional recombinant TcAAD from *T. camphorata* for biochemical characterization. The mature coding sequence of the TcAAD cDNA was introduced into an *S. cerevisiae* expression system and the resulting active enzyme was purified and characterized. Understanding the properties of this TcAAD will be beneficial for its potential in xenobiotic detoxification or production of natural flavors.

## Methods

### Total RNA preparation from *T. camphorata* and cDNA synthesis

Fruiting bodies of *T. camphorata,* a fungus that occurs only in the inner cavity of an endangered tree *Cinnamonum kanehirai*, were obtained from Asian Nova Biotechnology Inc, Taiwan (http://www.asian-bio.com/). Fresh fruiting bodies (wet weight 10 g) were frozen in liquid nitrogen and ground to powder in a ceramic mortar. PolyA mRNA (25 μg) was prepared using Novagen’s Straight A^’^s mRNA Isolation System (Gibbstown, NJ, USA). Four micrograms of the mRNA were used in the 5′-RACE-Ready cDNA and 3′-RACE-Ready cDNA synthesis using Clontech’s SMART RACE cDNA Amplification Kit (Mountain View, CA).

### Isolation of TcAAD cDNA

We have previously established an EST database from fruiting bodies of *T. camphorata* and sequenced all clones with insert size greater than 0.4 kb (data not shown). The identity of a partial AAD cDNA clone was assigned by comparing the inferred amino acid sequence in various databases using the basic local alignment search tool (BLAST) (http://www.ncbi.nlm.nih.gov/blast/Blast.cgi). Using the *T. camphorata* 5′-RACE-Ready cDNA as a template, an UPM primer (universal primer A mix, purchased from BD Biosciences, Palo Alto, CA) and a primer (5′GAT ATT CCA CTC GGC CTT GAC C3′ ) based on AAD partial cDNA, a 700 bp fragment was amplified by PCR. The 700 bp fragment was subsequently subcloned and sequenced. Based on the DNA sequence, a reverse primer AAD-1 (5′ CCT CTC GAG GGA GAT TCA A 3′) and a forward primer AAD-2 (5′GAG GTG ATG CTG GGG AAT3′) were synthesized. Using the *T. camphorata* 5′-RACE-Ready cDNA as a template and AAD-1 and UPM primer pair, a 400 bp fragment was amplified by PCR. Using the *T. camphorata* 3′-RACE-Ready cDNA as a template and AAD-2 and UPM primer pair, a 1,200 bp fragment was amplified by PCR. Both DNA fragments were subcloned into pCR4.0 vector and transformed into *E. coli* TOPO10, separately. The nucleotide sequence of the inserts was determined in both strands. Sequence analysis revealed that the combined sequences (400, 700 and 1,200 bp) covered an open reading frame of a putative AAD cDNA (1299 bp, GenBank no. HQ453361). The identity of this TcAAD clone was assigned by comparing the inferred amino acid sequence in various databases using the basic local alignment search tool (BLAST).

### Bioinformatics analysis of TcAAD

The BLAST program was used to search homologous protein sequences in the nonredundant database (NRDB) at the National Center for Biotechnology Information, National Institutes of Health (http://www.ncbi.nlm.nih.gov/). Multiple alignments were constructed using ClustalW2 program. Protein secondary structure was predicted by SWISS-MODEL program and represented as α helices and β strands. A 3-D structural model of TcAAD was created by SWISS-MODEL (Arnold et al., 2006) (http://swissmodel.expasy.org/) based on the known crystal structure of voltage-dependent potassium channels (PDB code:3EAU).

### Subcloning of TcAAD cDNA into an *E. coli* and yeast expression vector

The coding region of the TcAAD cDNA was amplified using gene specific flanking primers. The 5′ upstream primer contains *Eco* RI recognition site (5′GAA TTC GAT GTC CGT GGA GAA GAA GTC3′) and the 3′ downstream primer contains *Hind* III recognition site (5′AAG CTT AGA ATG GCC ATC GGC CAA C 3′). Using 0.2 μg of TcAAD cDNA as a template, and 10 pmole of each 5′ upstream and 3′ downstream primers, a 1,044 bp fragment encoding the putative mature TcAAD gene was amplified by PCR, and was ligated with cloning vector pCR4.0 and transformed into *E. coli*. The plasmid was isolated and digested with *Eco* RI and *Hind* III. The digestion products were separated on a 1.0% agarose gel. The 1,044 bp insert DNA was gel purified and subcloned into *Eco* RI and *Hind* III site of pET-20b(+) expression vector (Novagen, Darmstadt, Germany). The recombinant DNA was then transformed into *E. coli* C43(DE3). The recombinant protein was not overexpressed in the *E. coli* expression system.

Therefore the TcAAD gene was subcloned into a yeast expression system for overexpression. The coding region of the TcAAD cDNA was re-amplified by using two gene-specific primers: the 5′ upstream primer contains *Eco* RI recognition site (5′GAA TTC GAT GTC CGT GGA GAA GAA GTC3′) whereas the 3′ downstream primer contained a His6-tag and *Eco* RI recognition site (5′ CGT CTC GAA TTC TCA GTG GTG GTG GTG GTG GTG 3′). Using the 0.2 μg recombinant DNA of pET-20b(+)-TcAAD as a template, and 10 pmole of each 5′ upstream and 3′ downstream primers, a 1.0 kb fragment was amplified by PCR. The fragment was ligated into pCR4.0 and transformed into *E. coli*. The recombinant plasmid was isolated and digested with *Eco* RI. The digestion products were separated on a 1.0% agarose gel. The 1.0 kb insert DNA was gel purified and subcloned into the *Eco* RI site of the pYEX-S1 expression vector (Clontech, Mountain View, CA, USA) and introduced into *Saccharomyces cerevisiae* (trp^-^ ura^-^). The transformed yeast cells were selected by YNBDT (0.17% yeast nitrogen base, 0.5% ammonium sulfate, and 2% glucose) agar plates containing 20 μg Trp/mL. The presence of TcAAD gene in the selected transformants was verified by PCR using gene-specific flanking primers. The recombinant TcAAD protein was expressed in yeast in YPD medium (1% yeast extract, 2% peptone, 2% glucose). Overexpression of the functional recombinant TcAAD was analyzed by enzyme activity assay.

### Expression and purification of the recombinant TcAAD

The yeast transformant which containing the TcAAD gene was grown at 30°C, 170 rpm in 250 mL of YPD medium for 2 days. The cells were harvested and the soluble proteins extracted in PBS (phosphate buffer saline) with glass beads as described previously (Ken et al., 2006). The recombinant TcAAD was purified by Ni-NTA affinity chromatography (elution buffer: 30% PBS containing 20–250 mM imidazole) according to manufacturer’s instruction (Qiagen). The purified protein was analyzed by a 12% SDS-PAGE followed by staining with Coomassie Brilliant Blue R-250 and destaining. Protein concentration was determined by a Bio-Rad Protein Assay Kit (Richmond, CA) using bovine serum albumin as a standard (Bradford, 1976).

### Molecular mass analysis via JOEL MALDI-TOF (JMS-S3000, Japan)

The purified recombinant TcAAD (1 mg/mL) was dissolved in 0.3% PBS containing 0.05 mM imidazole and 0.45% glycerol. The sample (5 μL) was used for molecular mass determination using JOEL MALDI-TOF.

### TcAAD activity assay

The AAD activity was determined by measuring NADPH-dependent reduction of veratraldehyde (3,4-dimethoxybenzaldehyde, (CH_3_O)_2_C_6_H_3_CHO) at pH 6.0 (Muheim et al., 1991; Guillen and Evans, 1994)**.** A typical 100 μL reaction mixture contained 25 mM bis-tris- propane/HCl (pH 6.0), 0.2 mM NADPH and 0.2 mM veratraldehyde. The reaction was initiated by addition of 3 μg TcAAD. The reaction was followed by a decrease in *A*_365_ due to the oxidation of NADPH. A_365_ was used instead of A_340_ to reduce the interferences with the maximum absorbance of veratraldehyde at A_310_ (Guillen and Evans, 1994). Under the same conditions, another set of reactions was set up except that NADPH was replaced with NADH for enzyme activity assay. The molar absorption coefficient of NADH at 355 nm is 4390 M^-1^ cm^-1^.

The ability of the TcAAD to oxidize benzyl alcohols (benzyl alcohol; 2,4-dimethoxy benzyl alcohol; 3,4-dimethoxybenzyl alcohol and 4-(hydroxymethyl)benzoic acid) was tested by measuring NAD^+^-dependent oxidation of these benzyl alcohols to their corresponding benzylaldehydes by increasing the production of NADH at A_355_ nm. A typical 100 μL reaction mixture contained 50 mM glycine/NaOH (pH 9.6), 4 mM NAD^+^ and 4 mM benzyl alcohols (Siljegovic et al., 1998). The reaction was initiated by addition of 10 μg TcAAD. The reaction was followed by an increase in *A*_355_ due to the reduction of NAD^+^. Under the same conditions, another set of reactions was set up except for NAD^+^ was replaced with NADP^+^ for enzyme activity assay.

### Kinetic studies

The kinetic properties of the TcAAD (3 μg) was determined by varying the concentrations of veratraldehyde (0.1 to 0.4 mM) with fixed amount of 0.2 mM NADPH. The change in absorbance at 365 nm was recorded for one min. The molar absorption coefficient of NADPH at 365 nm is 3.5 mM^-1^ cm^-1^. The *K*_M_, V_max_ and *k*_cat_ were calculated from Lineweaver-Burk plots.

### Enzyme characterization

The TcAAD enzyme was tested for stability in terms of its activity under various conditions. Aliquots of the TcAAD sample were treated as follows: (1) *Thermal effect*. Each enzyme sample (3 μg/12 μL) was heated to 58°C for 2, 4, 8 or 16 min. Temperatures which are greater than 58°C were also performed. (2) *pH effect*. Each enzyme sample (3 μg/12 μL) was adjusted to desired pH by adding a half volume of buffer with different pHs: 0.2 M citrate buffer (pH 4.0), 0.2 M phosphate buffer (pH 5.0, 6.0, 7.0, 8.0, or 9.0) or 0.2 M CAPS buffer (pH 10.0). Each sample was incubated at room temperature for 30 min. At the end of each treatment, TcAAD enzyme activity were checked at pH 6.0 in the presence NADPH or was subjected to 12% SDS-PAGE analysis as mentioned above.

## Results

### Cloning and characterization of a cDNA encoding TcAAD

**Figure 1:**
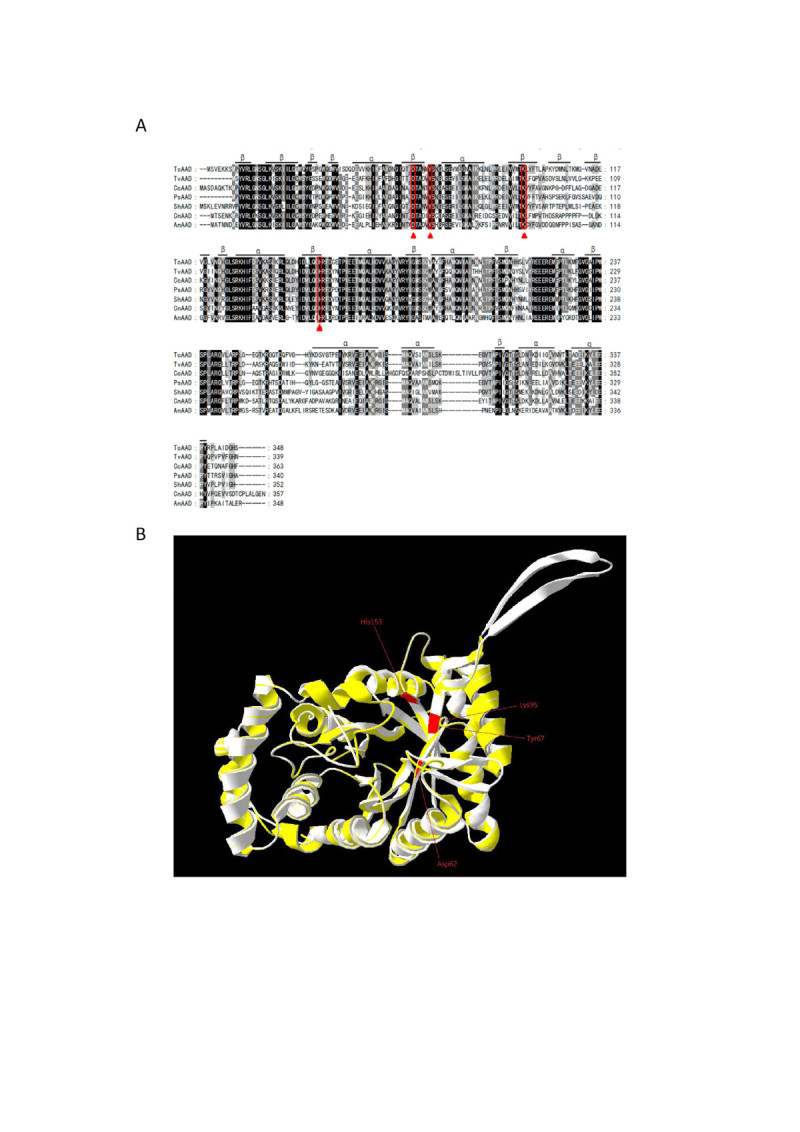
**Alignment of the amino acid sequences of TcAAD with other organism’s AADs, and its predicted 3-D structure. (A)** Sequence alignment: TcAAD (this study), TvAAD (*Trametes versicolor* FP-101664 SS1, EIW61070), CcAAD (*Coprinopsis cinerea* okayama7#130, XP_002911316), PsAAD (*Punctularia strigosozonata* HHB-11173 SS5, EIN04988), ShAAD (*Stereum hirsutum* FP-91666 SS1, EIM87563), CnAAD (*Cryptococcus neoformans* var. neoformans JEC21, XP_567886), CvAAD (*Coriolus versicolor*, AB070838). Protein secondary structure was predicted by SWISS-MODEL program and represented as α-helices and β-strands. The triangle indicates putative active site**. (B)** The structural model of TcAAD was predicted based on the known crystal structure of a shaker family voltage-dependent potassium channels (Kv1) subunit beta-2 from *Rattus norvegicus* (PDB code: 3EAU) via SWISS-MODEL program. TcAAD (light white) and template (light yellow, PDB code: 3EAU) was shown using protein solid ribbons. TcADD belongs to the aldo-keto-reductases superfamily with catalytic tetrad, Asp^62^, Tyr^67^, Lys^95^, and His^153^, which are conserved as they are present in almost all members of the superfamily (NCBI AKR superfamily conserved domain search). His^153^ is one of the catalytic tetrad and is the putative active site.

A putative TcAAD cDNA clone was identified on the basis of the consensus pattern and sequence homology to the published AADs in NCBI database. The entire coding region of TcAAD cDNA is 1,044 bp long and the deduced protein consists of 348 amino acid residues with calculated molecular mass of 39.1 kDa (GenBank no. HQ453361). Figure 1 shows the optimal alignment of the amino acid sequences of the TcAAD with 6 selected AAD sequences from other sources. This TcAAD shared 75% identity with TvAAD (*Trametes versicolor* FP-101664 SS1, EIW61070), 72% identity with CcAAD (*Coprinopsis cinerea* okayama7#130, XP_002911316), 69% identity with PsAAD (*Punctularia strigosozonata* HHB-11173 SS5, EIN04988), 65% identity with ShAAD (*Stereum hirsutum* FP-91666 SS1, EIM87563), 61% identity with CnAAD (*Cryptococcus neoformans* var. neoformans JEC21, XP_567886), and 29% identity with CvAAD (*Coriolus versicolor*, AB070838). The protein belongs to the aldo-keto reductase (AKR) superfamily. The AKR superfamily is one of the three enzyme superfamilies that perform oxidoreduction on a wide variety of substrates (Hyndman et al., 2003). AKRs play an important role in the phase II detoxification of a large number of pharmaceuticals, drugs, and xenobiotics. The highly conserved His^153^ is the putative catalytic site (Figure 1A, triangle) (Ichinose et al., 2002) and along with another three highly conserved amino acid residues, Asp^62^, Tyr^67^, and Lys^95^ are the catalytic tetrad in the putative active site pocket (see discussion session for details). The secondary structure, predicted by SWISS-MODEL program, showed 10 α helices and 14 β strands. The 3-D structural model superimposed with crystal structure of voltage-dependent potassium channels (PDB code:3EAU) (light yellow) via the SPDBV_4 program was shown using protein solid ribbon (Figure 1B).

### Expression and purification of the recombinant TcAAD

**Figure 2:**
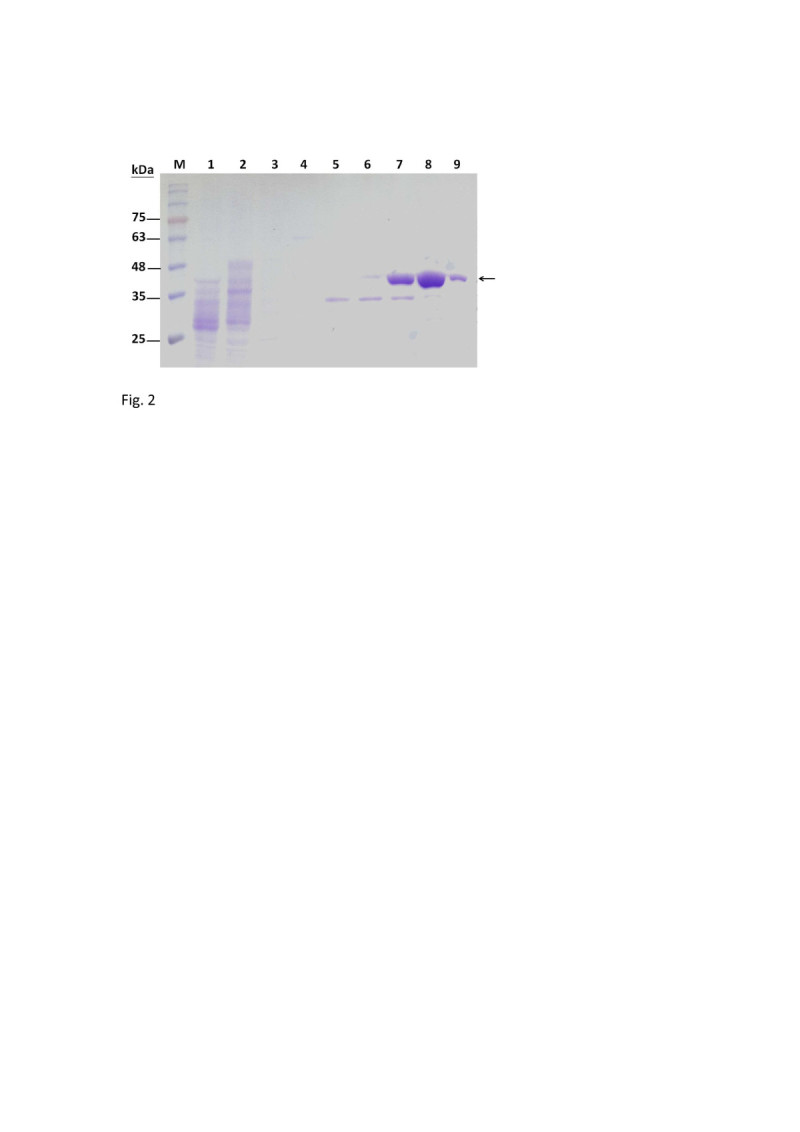
**Expression and purification of recombinant TcAAD in** ***Saccharomyces cerevisiae.*** Fifteen μL of each fraction was loaded into each lane of the 12% SDS-PAGE followed by Coomassie Brilliant Blue R-250 staining. Lane 1, crude extract from *Saccharomyces cerevisiae* expressing TcAAD; 2, flow-through proteins from the Ni-NTA column; 3–9, TcAAD eluted from Ni-NTA column. Molecular masses (in kDa) of standards are shown at left.

The coding region of the TcAAD was amplified by PCR and subcloned into an expression vector, pYEX-S1, as described in the Materials and methods. Positive clones were verified by DNA sequence analysis. The recombinant TcAAD was expressed, and the proteins were analyzed by a 12% SDS-PAGE in the absence of reducing agent and without boiling (Figure 2). The recombinant TcAAD was expressed as a His6-tagged fusion protein and was purified by affinity chromatography with nickel chelating Sepharose. A major band with molecular mass of ~39 kDa (expected size of TcAAD monomer) was detected in Ni-NTA eluted fractions by SDS-PAGE (Figure 2, lanes 8–9). The fractions contained pure protein were pooled and characterized further. Analysis of the TcAAD by MALDI-TOF confirms the presence of a single protein with molecular mass of 40.58 kDa (containing His6-tag). This indicates that the enzyme is predominantly monomeric in nature. The yield of the purified His6-tagged TcAAD was 1 mg from 250 mL of culture. Functional recombinant TcAAD was detected by enzyme activity assay as describe below.

### Kinetic studies of the purified TcAAD

**Figure 3:**
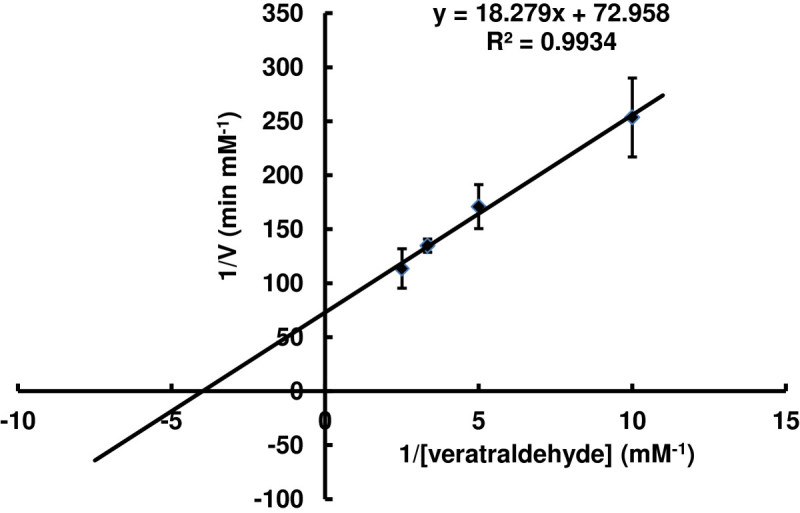
**Double-reciprocal plot of varying veratraldehyde on TcAAD activity.** The initial rate of the enzymatic reaction was measured at 0.2 mM NADPH with the veratraldehyde concentration varied from 0.1 to 0.4 mM. The *K*_M_ , V_max_ and *k*_cat_ were calculated from Lineweaver-Burk plots.

**Figure 4:**
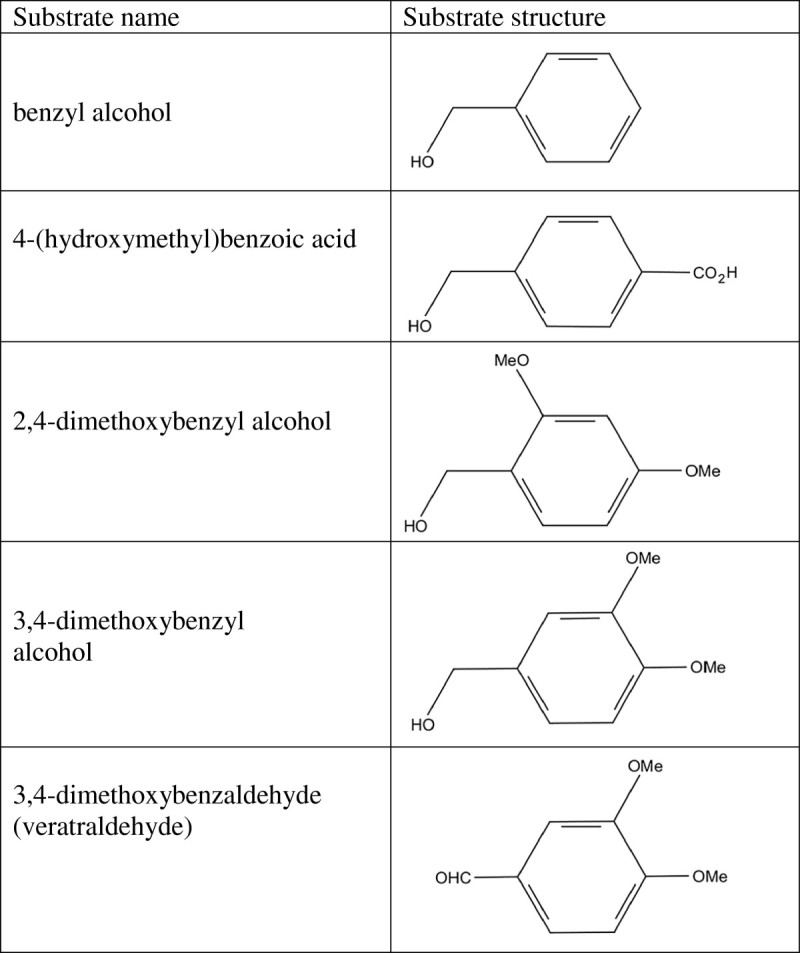
**Chemical structures of substrates**.

As shown in Figure 3, the Lineweaver-Burk plot of the velocity (1/V) against 1/[veratraldehyde] gave the *K*_M_ = 0.25 mM, V_max_ = 0.014 mM/min. The ability of TcAAD to oxidize other benzyl compounds was tested by monitoring the oxidation rate using 4 different alcohol compounds (benzyl alcohol, 3,4-dimethoxybenzyl alcohol, 2,4-dimethoxybenzyl alcohol and 4-(hydroxymethyl)benzoic acid ) via an increase in *A*_355_ due to the reduction of NAD^+^. As shown in Table 1, 4-(hydroxymethyl) benzoic acid was the best substrate for TcAAD among the benzyl alcohols tested (substrate structures as shown in Figure 4).

**Table 1:** **Summary of the reactions catalyzed by TcAAD**

Substrate	Cofactor	Product	V (nmole of NADH/min/mg of TcAAD)
benzyl alcohol	NAD^+^	benzyl aldehyde	262.72 ± 13.15 at pH 9.6
3,4-dimethoxybenzyl alcohol	NAD^+^	veratraldehyde	290.05 ± 13.15 at pH 9.6
2,4-dimethoxybenzyl alcohol	NAD^+^	2,4-dimethoxybenzyl aldehyde	232.35 ± 12.05 at pH 9.6
4-(hydroxymethyl)benzoic acid	NAD^+^		1099.47 ± 17.05 at pH 9.6
4-(hydroxymethyl)benzoic acid	NADP^+^		No reaction at pH 9.6
veratraldehyde	NADH	3,4-dimethoxybenzyl alcohol	No reaction at pH 6
veratraldehyde	NADPH	3,4-dimethoxybenzyl alcohol	V_max_ = 0.014 mM/min at pH 6

Enzymatic activity (V ± SD) was measured as described in Materials and Methods in the presence of 10 μg/0.1 mL enzyme, 4 mM NAD^+^, 0.1 mM alcohol related substrates at pH 9.6. The V was measured as the increase of NADH (355 nm) in the first 0.5-1 min reaction at 25°C. The reactions with veratraldehyde substrate were carried out in the presence of 3 μg/0.1 mL enzyme, 0.2 mM NADH or 0.2 mM NADPH and 0.2 mM veratraldehyde at pH 6. The V was measured as the decrease of NADH (355 nm) or NADPH (365 nm) in the first 0.5-1 min reaction at 25°C.

### Characterization of the purified TcAAD

The functional recombinant TcAAD enzyme expressed by yeast was shown to possess AAD activity by its ability to reduce veratraldehyde which is a benzyl aldehyde to its corresponding benzyl alcohol in the presence of NADPH at pH 6.0. In the reduction of veratraldehyde to its corresponding alcohol (3,4-dimethoxylbenzyl alcohol) at pH 6.0 and in the presence of NADPH, the conversion of NADPH to NADP^+^ is veratraldehyde concentration dependent. And the data were used for double-reciprocal plot. Under the same experimental conditions when NADPH was replaced with NADH, no enzyme activity was observed even when the substrate concentration of veratraldehyde was increased.

When benzyl alcohol; 2,4-dimethoxybenzyl alcohol; 3,4-dimethoxybenzyl alcohol and 4-(hydroxymethyl)benzoic acid were used as substrates to assay TcADD enzyme activity in the presence of NADP^+^ at pH 9.6, no enzyme activity was detected even when the substrate concentration increased. However, under the same reaction conditions when NADP^+^ was replaced with NAD^+^, TcADD enzyme activity was detected.

The recombinant TcAAD enzyme expressed by yeast was shown to possess AAD activity by its ability not only to reduce benzyl aldehyde via NADPH at pH 6.0 but also to oxidize benzyl alcohol via NAD^+^ at pH 9.6. It is interesting to note that reduction of benzyl aldehyde by the TcAAD at pH 6.0 is NADPH-dependent while NADH did not yield detectable activity under the same conditions (results not shown). Oxidation of benzyl alcohol by the TcAAD at pH 9.6 is NAD^+^-dependent (Table 1) while NADP^+^ did not yield detectable activity under the same conditions (results not shown).

**Figure 5:**
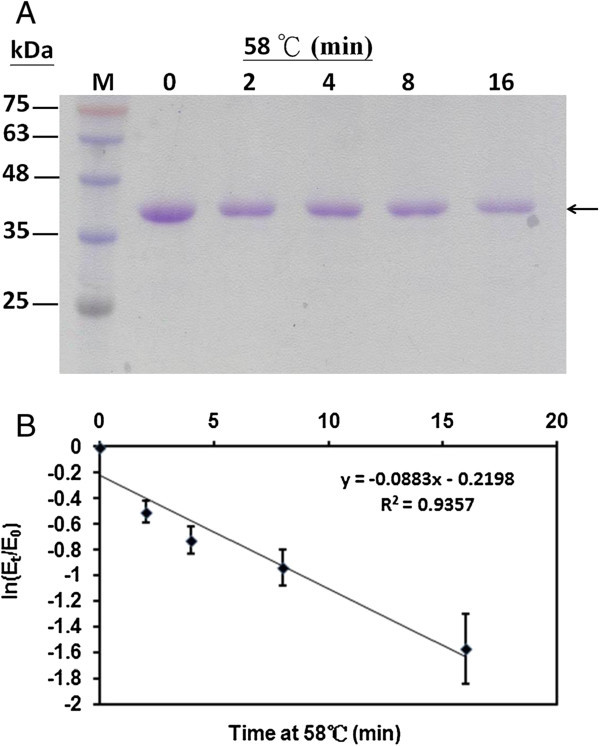
**Effect of temperature on the purified TcAAD.** The enzyme sample was heated at 58°C for various time intervals. Aliquots of the sample were taken at 0, 2, 4, 8 or 16 min analyzed by SDS-PAGE **(A)** and assayed for AAD activity. The thermal inactivation kinetics of AAD activity was plotted **(B)**. E_0_ and E_t_ are original activity and residual activity after being heated for different time intervals. Data are means of three experiments.

Heat stability of the TcAAD was tested to examine the effect of heat on the AAD activity, the purified TcAAD was heat-treated at 58°C for various time followed by TcAAD enzyme activity assay as described in “Materials and methods”. The TcAAD^’^s half-life of deactivation at 58°C was 4.2 min, and its thermal inactivation rate constant *k*_d_ was 7.4 x 10^-2^ min^-1^ (Figure 5). There was no detectable TcADD enzyme activity when it was heated above 60°C.

**Figure 6:**
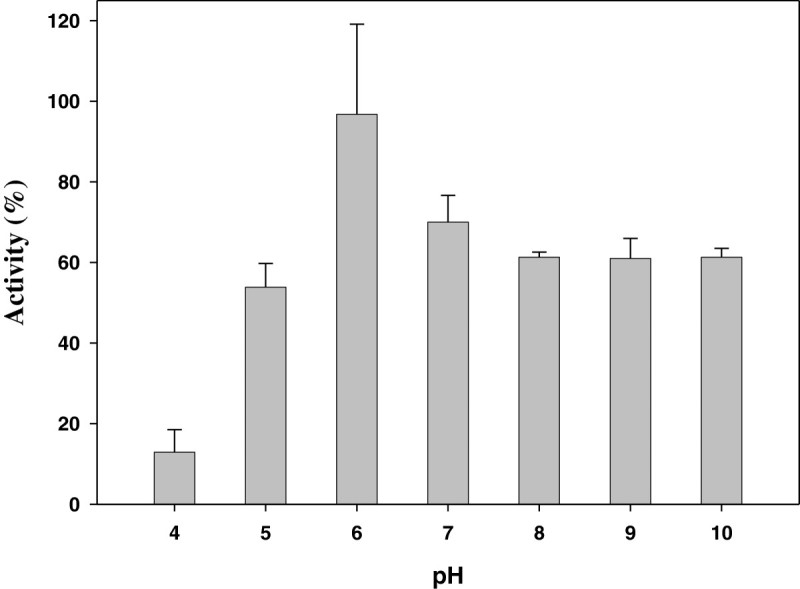
**Effect of pH on the purified TcAAD.** The enzyme samples were incubated with different pH buffer at 37°C for 30 min and then assayed for AAD activity. Data are means ± S.D. of three similar experiments.

With regard toTcAAD enzyme activity at different pH, the optimal activity to convert veratraldehyde to its corresponding 3,4-dimethoxybenzyl alcohol was observed at pH 6.0. As pH increased to pH 8 and 10, the enzyme retained 62% activity; and the enzyme retained 13% activity at pH 4 (Figure 6).

## Discussion and conclusions

This is the first report of TcAAD from *T. camphorata,* including the cloning, expression and purification of the functional recombinant enzyme from *Saccharomyces cerevisiae* for biochemical characterizations. The TcAAD enzyme behaves differently from other known AADs (discussed in the next paragraph) in the cofactors used. The enzyme can catalyze conversion of veratraldehyde to its corresponding benzyl alcohol (3,4-dimethoxybenzyl alcohol) at various pH from 4–10, with the optimal pH of 6.0 (Figure 6) and used NADPH as cofactor. When using NAD^+^ as cofactor at the optimal pH of 9.6, it can aslo catalyze the conversion of benzyl alcohols (benzyl alcohol; 2,4-dimethoxybenzyl alcohol; 3,4-dimethoxybenzyl alcohol; and 4-(hydroxymethyl)benzoic acid) to its corresponding benzylaldehydes (no activity was detected when NADP^+^ was used). Thus, as shown in Table 1 and Figure 6, the TcAAD’s optimal pH is at 6. It is possible that in *T. camphorata’s* the TcAAD functions in the reductive state using NADPH as cofactor*.*

Several NAD(P)^+^/NAD(P)H-dependent AAD enzymes from various bacterial & fungal sources have been reported: a) NAD^+^-dependent AAD enzyme from *Pseudomonas putida* was reported to oxidize benzyl alcohol derivatives (including 4-methoxybenzyl alcohol) to their corresponding benzylaldehydes at pH 9.6 (Siljegovic et al., 1998). b) NADPH-dependent AAD enzyme from *Pleurotus eryngii* was able to reduce benzylaldehyde derivatives (including veratraldehyde) to their corresponding benzyl alchhols at pH 6.0 (Guillen and Evans, 1994). c) NAD(P)H-dependent benzyl alcohol dehydrogenase activity was also reported from *Lactobacillus plantarum* WCFS1. This enzyme is more active in the presence of NAD^+^ than NADP^+^ in oxidizing benzyl alcohol, nerol, geraniol, phenethyl alcohol, cinnamyl alcohol, and coniferyl alcohol with optimal pH of 5.0 (Landete et al., 2008). d) Aryl-alcohol dehydrogenase from the white-rot fungus *Phanerochaete chrysosporium* strain BKM-F-1767 is more active in the presence of NADPH (*K*_M_ is 39 μM) than NADH (*K*_M_ is 220 μM) in reducing benzylaldehyde to benzyl alcohol (Yang et al., 2012). All the assay conditions for AADs activity have something in common. The reduction reactions were carried out at pH 6.0 with substrate concentration of benzylaldehyde in the range of 0.2-0.4 mM and enzyme amount about 3 μg, while higher concentrations of substrate of benzyl alcohols (4–5 mM) and enzyme amount (~10 μg) were used for the oxidation reactions at pH 9.6 (except for the *Lactobacillus plantarum* at pH 5.0). The substrate concentrations and the amount of TcAAD used in our enzyme-catalyzed assays were in the same range.

It is unclear why the TcADD uses different cofactors for the oxidation reactions (NAD^+^) and reduction reactions (NADPH) of substrates. In literature, structures of a large number of NAD^+^-binding enzymes have been studied and compared. It was concluded that these enzymes possess a similar structure with a β-α-β fold characteristic of nucleotide binding proteins (Schade et al., 1990). The analysis revealed a consensus sequence for the NAD^+^-binding dinucleotide fold (GXGXXG) which locates between the first β-strand and the α-helix allowing the formation of a tight turn (Wierenga et al., 1986)*.* Other analysis revealed a consensus sequence for the NADP^+^-binding dinucleotide fold (GXGXXAXXXAXXXXXXG) (Hanukoglu and Gutfinger, 1989). A search for NAD^+^ and NADP^+^-binding dinucleotide folds within the protein sequence of TcADD, we found conserved putative binding motifs for both. A putative NAD^+^-binding dinucleotide fold was found at positions 231–235 with the sequence GVGAI, and a putative NADP^+^-binding dinucleotide fold was found at positions 231–250 with the sequence GVGAIPWSPLARGVLARPLG.

Protein multiple sequence alignment indicated that TcAAD is a member of the NADP^+^ -dependent aldo-keto reductases (AKRs) superfamily having conserved domain with typical catalytic tetrad (four highly conserved amino acid residues, Asp, Tyr, Lys and His interact with the substrate and NADP^+^). Indeed, the catalytic tetrad, Asp^62^, Tyr^67^, Lys^95^, and His^153^ are present in the TcAAD sequence. Regardless that members of the AKRs superfamily have different 3-D structures, they have a common conserved domain consisted of 8 parallel β-strands in the center surrounded by 8 α-helices to form a (αβ)_8_-barrel which contains a NADP^+^-binding motif (NCBI AKRs superfamily conserved domain search. http://www.ncbi.nlm.nih.gov/Structure/cdd/wrpsb.cgi). The domain structure is arranged in that the nicotinamide-binding half (3 parallel β-strands and 2 α-helices) is structurally similar to the adenine-binding half (another 3 parallel β-strands and 2 α-helices) to form a Rossmann fold for NADP^+^-binding.

Using the conserved domain search tool, we found a total of 25 conserved residues in the putative active site pocket of the TcAAD, including the catalytic tetrad. These residues are Gly^26^, Thr^27^, Met^28^**,** Asp^62^, Tyr^67^, Lys^95^, His^153^, Arg^154^, Ser^183^, Ser^184^, Gln^209^, Trp^237^, Ser^238^, Pro^239^, Leu^240^, Ala^241^, Arg^242^, Ala^290^, Ile^307^, Val^308^, Gly^309^, Thr^310^, Asn^315^, Asp^318^, and Ile^319^.

TcAAD is a member of the aldo-keto reductases superfamily which prefers NADPH over NADH (Barski et al., 2008). Therefore, it is logical to explain the requirement of NADPH of TcAAD to catalyze reduction reactions at pH 6.0. However we can’t explain its novelty in which the enzyme does not utilize NADP^+^ to oxidize its substrates at pH 9.6, instead NAD^+^ was used. It has been reported that the active site pocket of aldose reductases is hydrophobic in nature and favors aromatic and nonpolar substrates over polar ones (Wilson et al., 1992). However, contradict to the above statement; the active site pocket of TcAAD contains more polar or charged residues under physiological condition (15 out of 25 amino acid residues). Maybe at pH 9.6, TcAAD exists in a conformation favors NAD^+^ over NADP^+^. At pH 6, TcAAD exists in another conformation favors NADPH over NADH.

The TcAAD may be considered in the design of metabolic engineering strategies/synthetic biology systems for biotechnological applications. For instance, TcADD can be used to degrade aromatic inhibitors that are present in lignocellulosic hydrolysates which will otherwise impair yeast fermentation (Almeida et al., 2007). Additionally, the enzyme may be used in production of natural flavors and fragrances such as the rose-like flavor compound 2-Phenylethanol (Yang et al., 2012).

## Authors’ original submitted files for images

Below are the links to the authors’ original submitted files for images. Authors’ original file for figure 1 Authors’ original file for figure 2 Authors’ original file for figure 3 Authors’ original file for figure 4 Authors’ original file for figure 5 Authors’ original file for figure 6

## Abbreviations

AAD: Aryl-alcohol dehydrogenases
SDS-PAGE: Sodium dodecyl sulfate-polyacrylamide gel electrophoresis
PBS: Phosphate buffer saline.
