# Supplementary figures and images for: Biochemical characterization of a functional recombinant aryl-alcohol dehydrogenase from Taiwanofungus camphorata
Source: Bot Stud. 2014 Feb 2;55:14. doi: 10.1186/1999-3110-55-14 (PMC5432818; doi:10.1186/1999-3110-55-14)

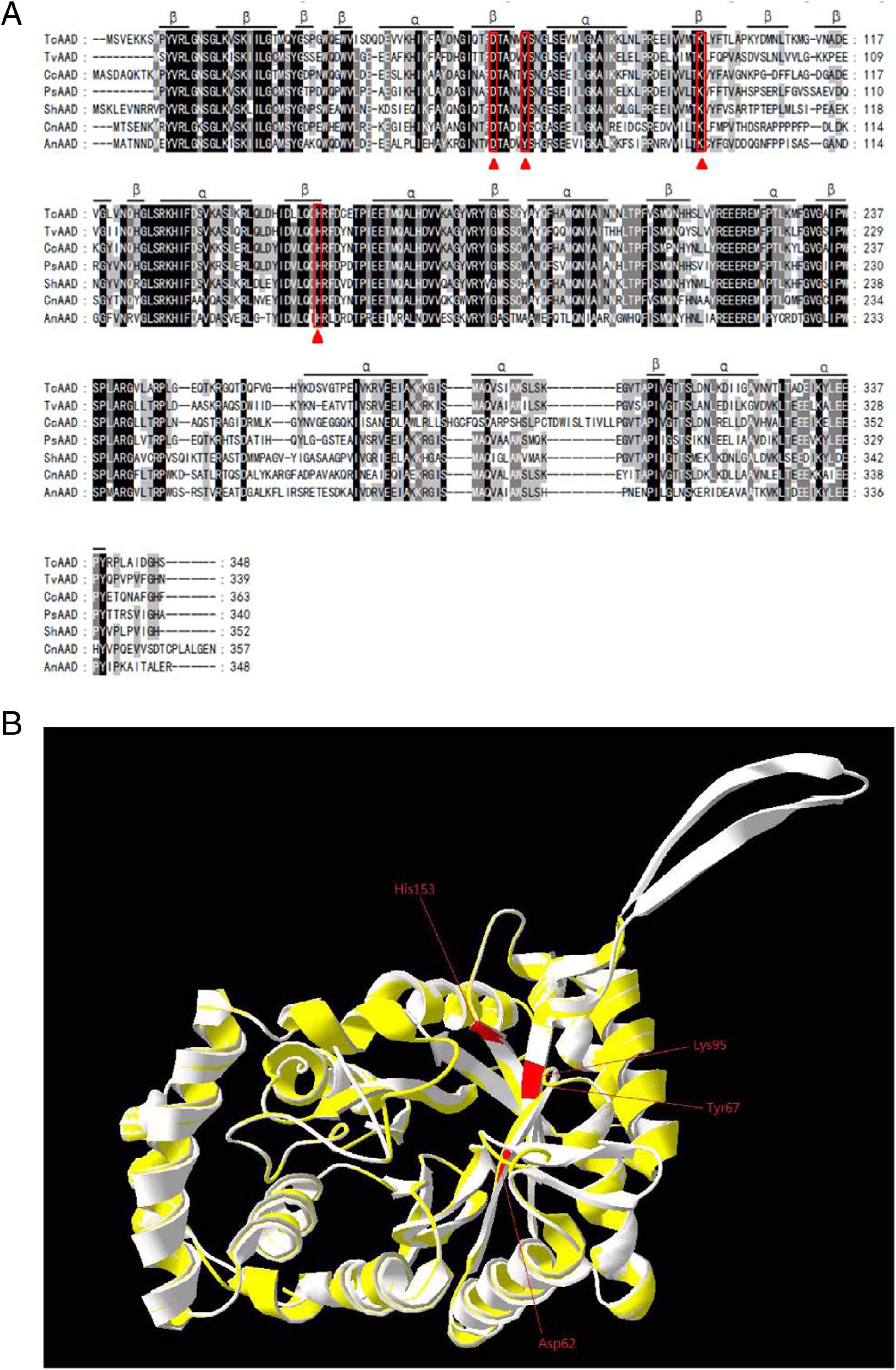

Supplement: Supplementary file 1 — Authors’ original file for figure 1 [file 40529_2013_63_MOESM1_ESM.tif]

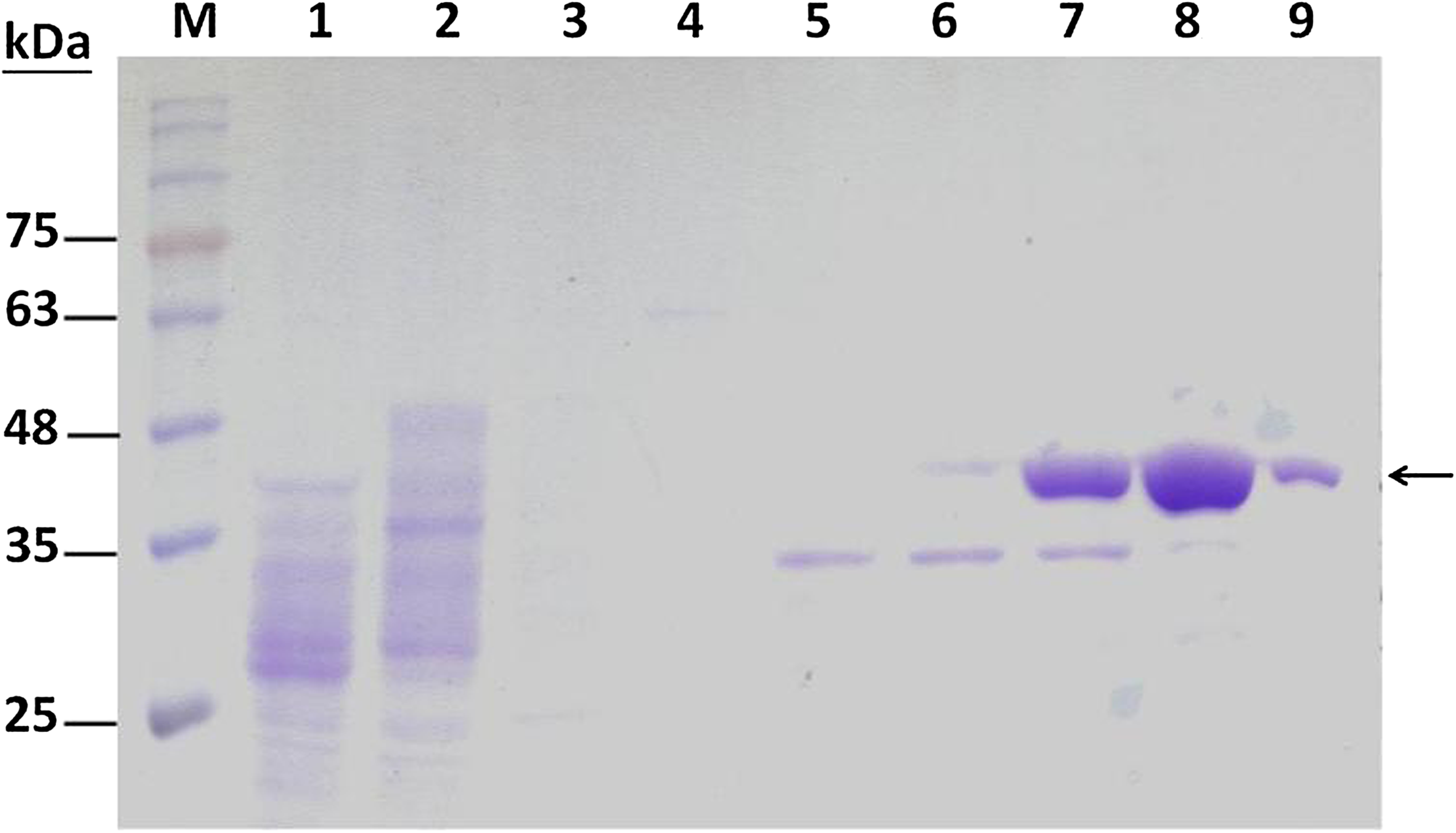

Supplement: Supplementary file 2 — Authors’ original file for figure 2 [file 40529_2013_63_MOESM2_ESM.tif]

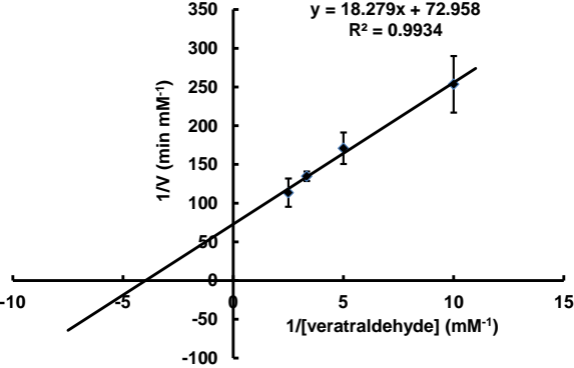

Supplement: Supplementary file 3 — Authors’ original file for figure 3 [file 40529_2013_63_MOESM3_ESM.pdf]

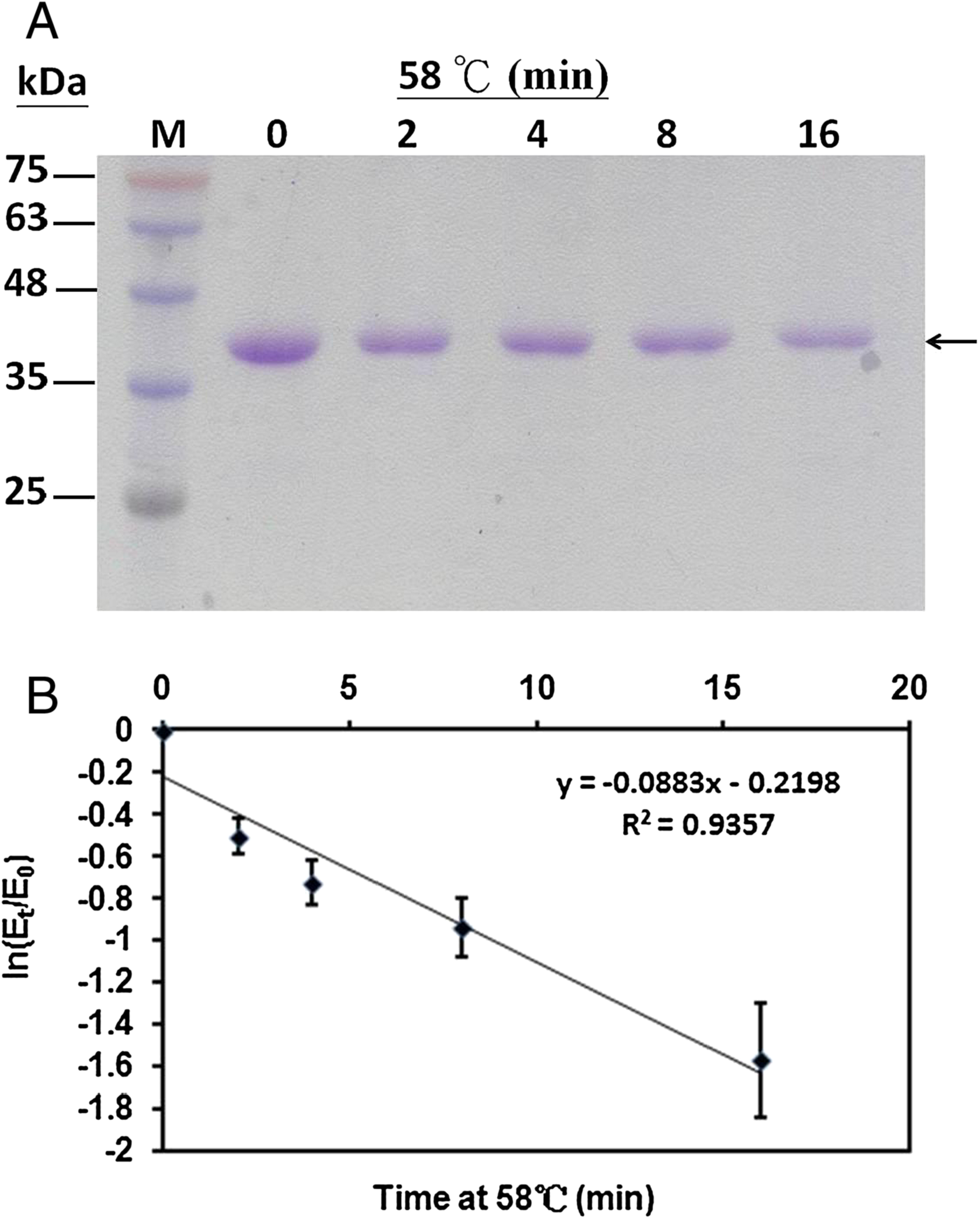

Supplement: Supplementary file 5 — Authors’ original file for figure 5 [file 40529_2013_63_MOESM5_ESM.tiff]

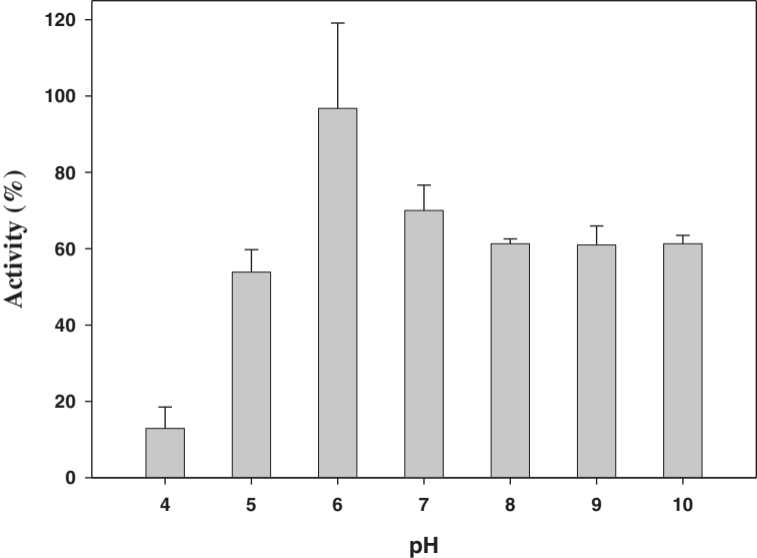

Supplement: Supplementary file 6 — Authors’ original file for figure 6 [file 40529_2013_63_MOESM6_ESM.pdf]
