# Supplementary material for: Biochemical characterization of a functional recombinant aryl-alcohol dehydrogenase from Taiwanofungus camphorata
Source: Bot Stud. 2014 Feb 2;55:14. doi: 10.1186/1999-3110-55-14 (PMC5432818; doi:10.1186/1999-3110-55-14)
Supplement: Supplementary file 4 — Authors’ original file for figure 4 [file 40529_2013_63_MOESM4_ESM.pdf]

Substrate name	Substrate structure
benzyl alcohol	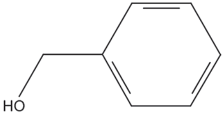
4-(hydroxymethyl)benzoic acid	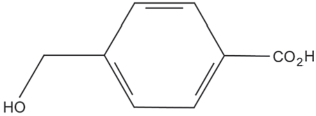
2,4-dimethoxybenzyl alcohol	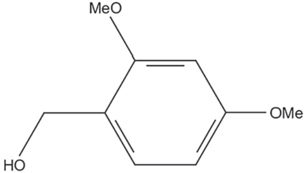
3,4-dimethoxybenzyl alcohol	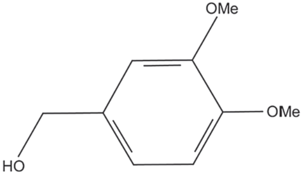
3,4-dimethoxybenzaldehyde (veratraldehyde)	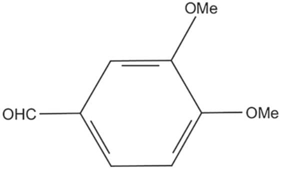
